# Increase in Lymphadenitis Cases after Shift in BCG Vaccine Strain

**DOI:** 10.3201/2109.150289

**Published:** 2015-09

**Authors:** Giorgi Kuchukhidze, Ana Kasradze, Tamar Dolakidze, David Baliashvili, Tsira Merabishvili, Henry M. Blumberg, Russell R. Kempker

**Affiliations:** National Center for Disease Control and Public Health, Tbilisi, Georgia (G. Kuchukhidze, A. Kasradze, T. Dolakidze, D. Baliashvili, T. Merabishvili);; Emory University School of Medicine, Atlanta, Georgia, USA (H.M. Blumberg, R.R. Kempker)

**Keywords:** BCG, lymphadenitis, vaccine, tuberculosis and other mycobacteria, Bacillus Calmette-Guérin, Georgia

**To the Editor:** Bacillus Calmette-Guérin (BCG) vaccine is one of the most commonly used vaccines for tuberculosis (TB) worldwide (*1*). The original BCG strain was developed in 1921. Numerous strains have since been developed, and 5 strains, including Danish SSI 1331 (Statens Serum Institute, Copenhagen, Denmark), account for >90% of BCG vaccine used. Each strain has unique characteristics and a different reactogenicity profile (*2*). The most common severe adverse events related to BCG vaccination are nonsuppurative and suppurative lymphadenitis.

In the country of Georgia, BCG vaccine is administered routinely to infants (estimated coverage 96%); the National Center for Disease Control and Public Health receives its vaccine supply from the United Nations Children’s Fund and is responsible for countrywide distribution. Before 2012, Russian BCG-I (Bulbio, Sofia, Bulgaria) and Danish SSI 1331 strains were used (≈50% each). Shortly after a change to exclusive use of the Danish 1331 strain during 2012–2013, an increasing number of BCG-related lymphadenitis cases were reported to the National Center for Tuberculosis and Lung Diseases (NCTLD). We aimed to quantify the increase in cases of BCG lymphadenitis and to evaluate clinical management of the cases. The Institutional Review Boards of Emory University (Atlanta, GA, USA) and the National Center for Disease Control and Public Health approved the study.

Medical chart abstraction was conducted for all infants with BCG lymphadenitis either reported to the NCTLD or found by inquiry of pediatricians at the largest children’s hospital in the country during January 2012–July 2013. We used national surveillance data to obtain the number of live-born infants.

BCG vaccine is given intradermally over the deltoid muscle on the left arm to infants within 5 days after birth at the maternity hospital. BCG lymphadenitis was clinically defined as ipsilateral axillary lymph node enlargement developing within 2 years after vaccination. If the patient was brought for care to the NCTLD, a sample was obtained through aspiration for acid-fast bacilli smear; culture; and, if necessary, drug-susceptibility testing (*3*). Although treatment was at the discretion of clinicians, national TB program treatment guidelines did not include management of BCG-related adverse events.

During 2007–2011, six cases of BCG lymphadenitis were reported to the NCTLD. During the 19-month study period, we found 23 cases of BCG lymphadenitis: 15 reported to the NCTLD and 8 diagnosed at the Tbilisi children’s hospital and ascertained by inquiry (Table). In all cases, a 0.05-mL dose of Danish SSI BCG vaccine (series 111003A and 111021A) was used. The 15 infants from the NCTLD were vaccinated at 8 maternity hospitals: 6 in Tbilisi and 2 in outside regions. A total of 14,230 live-born infants were registered at hospitals reporting BCG lymphadenitis in 2012. Based on the following calculation—16 cases/(14,230 live-born infants × 96% vaccination coverage)—the estimated prevalence of BCG-related suppurative lymphadenitis in 2012 was 1.12 cases per 1,000 infants.

**Table:** Characteristics of 23 infants with BCG lymphadenitis, Georgia, January 2012–July 2013*

Hospital, infant no.	Characteristic†							Treatment outcome‡
	Sex	Date of birth	Age at presentation, mo	Size of axillary lymph node, mm	Culture	Surgery	Drugs used	
NCTLD, n = 15								
1	F	2012 Jan 9	5	20	Pos	No	R, I, P	Completed
2	M	2012 Dec 19	5	14	ND	No	R, I, P	Completed
3	F	2012 Jul 2	9	40	NA	No	R, I, P	Completed
4	F	2012 May 31	12	28	NA	No	R, I, P	Completed
5	M	2012 Jul 25	4	15	NA	No	R, I, P	Computed
6	F	2012 Aug 16	1	15	NA	No	R, I, P	Defaulted
7	M	2012 Feb 7	2	22	NA	No	R, I, P	Completed
8	M	2011 Nov 28	3	24	NA	No	R, I, P	Defaulted
9	M	2012 Jul 9	2	18	NA	No	R, I, P	Defaulted
10	M	2012 Aug 7	7	23	NA	Yes	R, I, E	Unknown
11	M	2012 May 10	4	56	NA	No	R, I, P	Completed
12	M	2012 Nov 13	6	60	NA	No	R, I, P	Completed
13	M	2012 Oct 1	2	11	Pos	Yes	R, I	Completed
14	M	2012 Feb 22	9	24	NA	No	R, I, P, E	Completed
15	F	2012 Feb 24	5	15	NA	Yes	R, I, P	Complete
Pediatric hospital, n = 8								
1	F	2013 Jan 28	4	25	ND	Yes	None	Unknown
2	M	2012 Jun 15	8	20	ND	Yes	None	Unknown
3	M	2012 Mar 25	15	17	ND	Yes	None	Unknown
4	M	2012 Jan 28	6	21	ND	Yes	None	Unknown
5	M	2013 Jan 28	4	21	ND	Yes	None	Cured
6	M	2012 Jun 28	8	25	ND	Yes	None	Unknown
7	M	2013 Mar 28	5	15	ND	No	None	Cured
8	F	2013 Jun 8	1	17	ND	No	None	Cured

*BCG, bacillus Calmette-Guérin vaccine; POS, positive; E, ethambutol; I, isoniazid; P, pyrazinamide; R, rifampin; NA, not available; ND, not done; NCTLD, National Center for Tuberculosis and Lung Diseases. †For all patients, type of BCG strain used was Danish SSI (Statens Serum Institute, Copenhagen, Denmark). ‡Defaulted is an outcome definition applied by the tuberculosis program when a patient misses treatment for 2 consecutive months and is considered lost to follow-up.

Median time from BCG vaccination to onset of lymphadenitis was 5 months (range 1–15 months). No patients had systemic signs or symptoms.

After a change in BCG vaccine strains in Georgia to the exclusive use of BCG SSI vaccine, we found a substantial increase in the known prevalence of BCG-associated lymphadenitis. We found 23 cases of BCG-associated lymphadenitis during a 19-month period, ≈4 times the number of reported cases during the prior 5 years, when multiple vaccine strains were used. The estimated prevalence of suppurative lymphadenitis (1.12 cases/1,000 infants) was higher than the expected rate of <1/1,000 given by the manufacturer (*4*). Our rate is probably an underestimation, given a mainly passive system of surveillance. Prior studies in various countries have similarly shown increased BCG lymphadenitis with the introduction of the BCG SSI vaccine (*5*–*7*).

We found different approaches to treatment of BCG-associated lymphadenitis depending on where care was received. Physicians at the NCTLD prescribed first-line anti-TB medications, including pyrazinamide, whereas patients managed at the children’s hospital were treated with either surgical excision or a conservative watch-and-wait approach. Although no official treatment guideline exist for suppurative BCG-associated lymphadenitis, a recent meta-analysis found no benefit to using anti-TB medications (*8*). Furthermore, *Mycobacterium bovis* is inherently resistant to pyrazinamide. A randomized controlled trial found needle aspiration to improve rates and speed of healing of suppurative nodes and is the only evidence-based effective treatment (*9*). Surgical excision remains controversial because of potentially high rates of significant scarring (*10*). For nonsuppurative lymphadenitis, a watch-and-wait approach is recommended because most resolve rapidly (*8*).

Given our findings, the National TB Program in Georgia subsequently created a management protocol. This protocol recommends no intervention for nonsuppurative lymphadenitis and needle aspiration for suppurative local lymphadenitis.

In summary, we found an increasing rate of BCG-associated lymphadenitis after a shift to exclusive BCG SSI vaccine use in Georgia. Countries with a BCG vaccination policy should have a clear protocol on management of BCG vaccine–related adverse events to avoid inappropriate treatment in children.
